# Genome sequence data of multidrug-resistant *Enterococcus faecalis* HMTZ24 carrying multiple virulence factors, isolated from a urinary tract infection in Mosul, Iraq

**DOI:** 10.1016/j.dib.2025.112352

**Published:** 2025-12-03

**Authors:** Talal S. Salih, Zeyad T. Al-Rrassam, Muhammad A. Muhammad, Hozan R. Ziwar, Mohammed M. Mohammed

**Affiliations:** a Department of Medical Physics, College of Science, University of Mosul, Iraq; b Department of Biology, College of Science, University of Mosul, Iraq

**Keywords:** *Enterococcus faecalis*, Genome sequence, Phylogenomic, Sequence typing, Antimicrobial resistance, Mobile genetic elements, Virulence

## Abstract

*Enterococcus faecalis* is a common pathogen associated with urinary tract infections (UTIs) worldwide. Here we present the draft genome sequence of *E. faecalis* strain HMTZ24, isolated from the urine of a female patient in Mosul, Iraq. Whole-genome sequencing was performed on the Illumina NovaSeq 6000 platform. The assembled genome is 2,623,745 base pairs (bp) in length distributed across 128 contigs, with an N50 of 41,811 bp and a GC content of 37.73%. Annotation revealed 2,510 coding sequences (CDSs), 50 tRNAs, and 5 rRNA genes. Phylogenomic taxonomy analysis indicated that strain HMTZ24 is closely related to *E. faecalis* NBRC 100480 (=ATCC 19433) with a digital DNA-DNA hybridisation (dDDH) value of 92.9 % and an average nucleotide identity (ANI) of 99.16 %. Multilocus sequence typing (MLST) assigned the HMTZ24 strain to sequence type 28 (ST28). The genome harbors six antimicrobial resistance genes confirming resistance to nalidixic acid, ciprofloxacin, chloramphenicol, erythromycin, rifampin, trimethoprim, lincomycin, clindamycin, tetracycline, and vancomycin. Two mobile genetic elements (MGEs) including Tn6009 and ISLgar5, and 14 virulence factor genes including *ebpA, ebpB, ebpC, ace, strA, espfs, cad, camE, cCf10, cOB1, gelE, tpx, efaA*, and *ElrA* were also identified. The dataset provides a valuable genomic resource for comparative analyses of *E. faecalis* strains, supporting studies on antimicrobial resistance, virulence factors and regional epidemiology. The draft genome sequence of strain HMTZ24 has been deposited in NCBI under the accession number JBISBO000000000.1.

Specifications Table

**Table:** 

Subject	Biology
Specific subject area	Genomics, Molecular Microbiology, Bacteriology.
Type of data	Whole Genome Sequence Data in FASTA format, Analyzed, Tables, figures, deposited.
Data collection	Genomic DNA of *E. faecalis* HMTZ24 was extracted from a 24-hour culture grown on mannitol salt agar using the Bacterial DNA Isolation Kit. The DNA library was prepared using the TruSeq Nano DNA Library kit and sequenced on the Illumina NovaSeq 6000 platform (Illumina, USA). Raw reads were quality-trimmed using Trimmomatic v0.30, and the genome was de novo assembled with SPAdes v3.5. Annotation was carried out using the NCBI Prokaryotic Genome Annotation Pipeline (PGAP) v6.9. General assembly statistics were obtained with QUAST v5.3.0, while genome completeness was assessed using CheckM v1.2.3. Phylogenomic tree reconstruction and digital DNA-DNA hybridization (dDDH) analysis were performed using the Type Strain Genome Server (TYGS). Average nucleotide identity (ANI) was calculated with the OrthoANIu tool. Antimicrobial resistance genes were predicted using the Comprehensive Antibiotic Resistance Database (CARD v4.0.1) and ResFinder v4.7.2. Mobile genetic elements were identified with MGEfinder v1.0.3, and virulence factor genes were predicted using VirulenceFinder v2.0.
Data source location	*E. faecalis* strain HMTZ24 was isolated from a urinary tract infection case in Mosul, Iraq (36.3456° N, 43.1575° E).
Data accessibility	The draft genome sequence of *E. faecalis* strain HMTZ24 is available in the NCBI GenBank database. Data identification number: Genome accession: JBISBO000000000.1, BioProject accession: PRJNA1177504, BioSample accession: SAMN44427890 Direct URL to data: https://www.ncbi.nlm.nih.gov/nuccore/JBISBO000000000 https://www.ncbi.nlm.nih.gov/bioproject/PRJNA1177504 https://www.ncbi.nlm.nih.gov/biosample/SAMN44427890
Related research article	None*.*

## 1 Value of the Data

• This study provides a detailed genomic characterizes the genome of *E. faecalis* HMTZ24, isolated from a severe urinary tract infection, using a next generation sequencing.
• The dataset reveals key antimicrobial resistance and virulence genes, offering insights into predicted drug resistance phenotypes in *E. faecalis.*
• The genome sequence of *E. faecalis* HMTZ24 from Iraq provides valuable information for researchers investigating its phylogeny, pathogenicity, and comparative genomics relative to other publicly available strains in GenBank, and represents an important to establishing a local clinical database of pathogens in Iraq and neighboring countries.

## 2 Background

*Enterococcus faecalis* is a Gram-positive, non-spore-forming, facultative anaerobic bacterium and a common cause of urinary tract infections (UTIs), along with other bacterial species such as *Escherichia coli, Klebsiella pneumoniae, Proteus mirabilis*, and *Staphylococcus saprophyticus*, collectively responsible for millions of infections worldwide each year [1]. In recent years, clinical isolates have shown increased pathogenicity and antimicrobial resistance [2]. The genome of *E. faecalis* harbors diverse of mechanisms, including resistance genes, virulence factors and biofilm formation capabilities, which contribute to its pathogenicity and drug resistance [3]. Genomic analyses of UTIs pathogens from Iraq remain limited. Here, we present the draft genome sequence analysis of *E. faecalis* strain HMTZ24, isolated from the urine of a female patient in Mosul, Iraq, along with an assessment of its genome characteristics, phylogenomic relationships, sequence type, and the presence of antimicrobial resistance and virulence genes.

## 3 Data Description

**Fig. 1:**
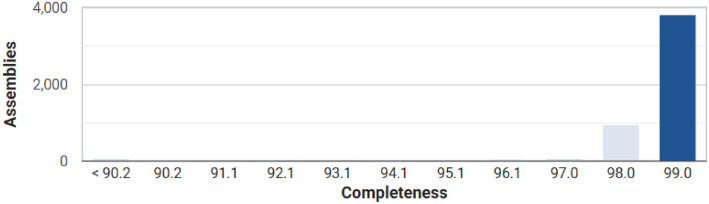
Genome completeness of *E. faecalis* HMTZ24 assessed with CheckM v1.2.3, using the Prokaryotic Genome Annotation Pipeline (PGAP) gene set and the *Enterococcus faecalis-*specific CheckM marker set.

The assembled genome of *E. faecalis* HMTZ24 has a total predicted length of 2,623,745 bp, distributed across 128 contigs, with an N50 of 41,811 bp and a GC content of 37.73 %. Genome annotation identified 2,569 genes, including 2,510 protein-coding sequences (CDSs), 50 tRNA genes, 5 rRNA genes, and 4 ncRNA genes. A detailed summary of the genome characteristics is presented in Table 1. CheckM analysis indicated 99.24 % genome completeness with a very low contamination level of 0.05 % (Fig. 1).

**Table 1:** General genome characteristics of *E. faecalis* HMTZ24.

Features	Value
Total length size (bp)	2,623,745
DNA GC content (%)	37.73
Genome coverage (X)	100
Number of contigs	128
Number of contigs (≥ 1000 bp)	109
Longest contig size (bp)	125,381
Shortest contig size (bp)	526
N50	41,811
N90	13,685
L50	20
L90	59
Genes (total)	2,569
CDSs (with protein)	2,470
CDSs (without protein)	40
tRNA	50
rRNA	3, 1, 2 (5S, 16S, 23S)
ncRNA	4

**Fig. 2:**
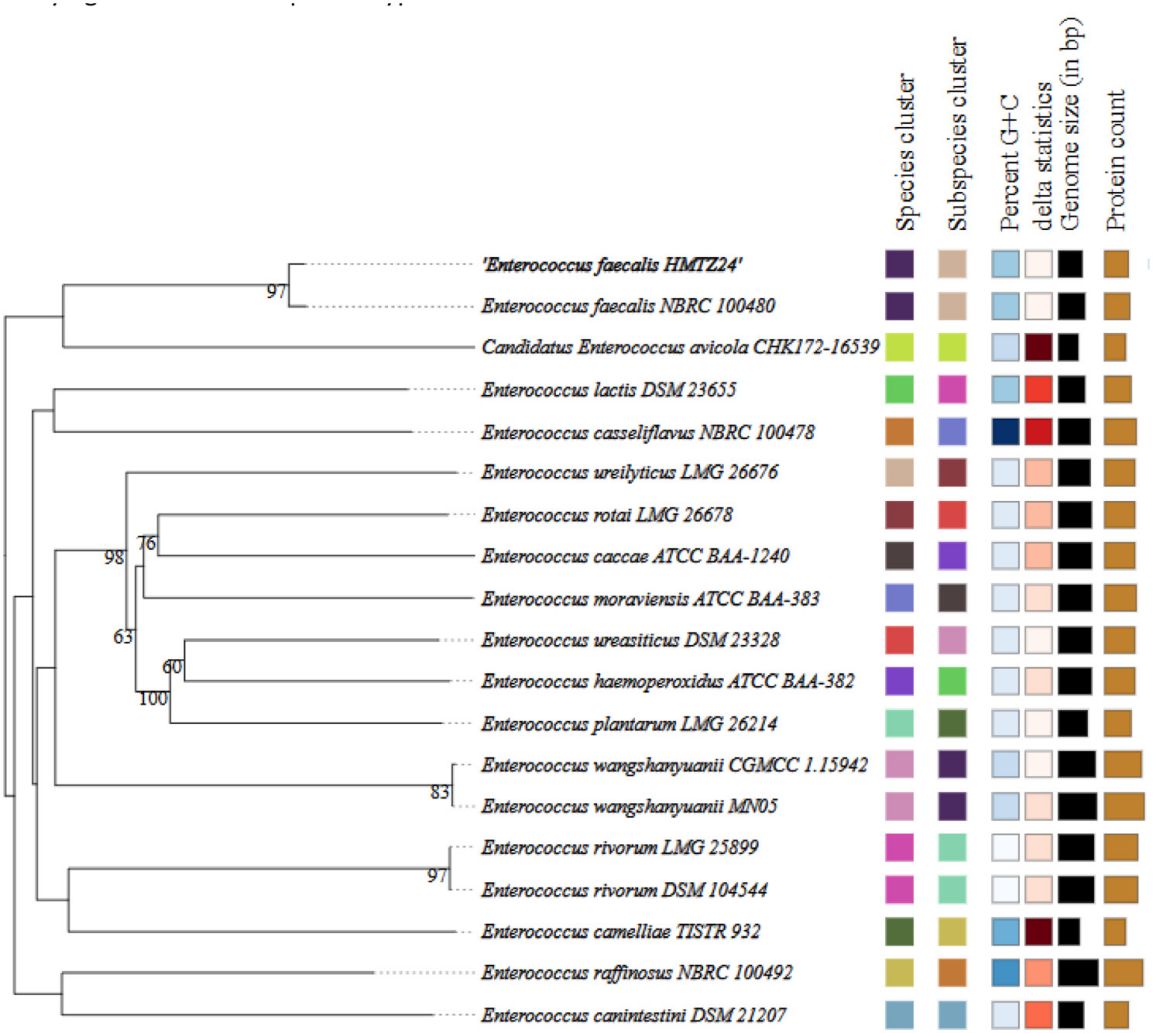
Phylogenomic tree of *E. faecalis* HMTZ24 (indicated in bold) and its closest related *Enterococcus* species, generated from the draft genome sequence using the TYGS platform with 100 × bootstrap support.

The whole-genome phylogenetic tree is shown in Fig. 2. Phylogenetic analysis revealed that *E. faecalis* HMTZ24 is most closely related to the type strain *E. faecalis* NBRC 100480 (=ATCC 19433), with a digital DNA-DNA hybridization (dDDH) value of 92.9 % and an average nucleotide identity (ANI) of 99.16 %. Multilocus sequence typing (MLST) of HMTZ24 identified seven loci (Table 2), classifying the strain as sequence type ST28.

**Table 2:** MLST profile of *E. faecalis* HMTZ24, assigned to sequence type ST28 based on the PubMLST database.

Locus	Identity	Length	Gaps	Allele
*aroE*	100	459	0	*aroE*_8
*gdh*	100	530	0	*gdh*_4
*gki*	100	438	0	*gki*_3
*gyd*	100	395	0	*gyd*_4
*pstS*	100	583	0	*pstS*_8
*xpt*	100	456	0	*xpt*_1
*yqiL*	100	436	0	*yqiL*_3

Seven antibiotic resistance genes were identified in the genome, conferring resistance to multiple drugs, including nalidixic acid, ciprofloxacin, chloramphenicol, erythromycin, rifampin, trimethoprim, lincomycin, clindamycin, tetracycline, and vancomycin (Table 3). Two mobile genetic elements (MGEs) associated with resistance, Tn6009 (an integrative conjugative element) and ISLgar5 (an insertion sequence), were detected (Table 4).

**Table 3:** Antibiotic resistance genes identified in the genome of *E. faecalis* HMTZ24 using the CARD v4.0.1 and ResFinder v4.7.2 databases.

Resistance gene	Drug class	Resistance mechanism	Predicted phenotype	Identity (%)	PMID
*gyrA*	fluoroquinolone	antibiotic target alteration	nalidixic acid, ciprofloxacin	99.8	12373497
*catA8*	phenicol antibiotic	antibiotic inactivation	chloramphenicol	99.53	9349809
*efrA*	macrolide antibiotic, rifamycin antibiotic	antibiotic efflux	erythromycin, rifampin	99.4	14638474
*dfrE*	diaminopyrimidine antibiotic	antibiotic target replacement	trimethoprim	98.78	9869579
*lsa(A)*	lincosamide	antibiotic target protection	lincomycin, clindamycin	98.53%	15616272
*tet(M)*	tetracycline antibiotic	antibiotic target protection	tetracycline	94.05	19475445
*vanT* gene in *vanG* cluster	glycopeptide antibiotic	antibiotic target alteration	vancomycin	35.44	16723588

**Table 4:** Mobile genetic elements (MGEs) identified in the genome of *E. faecalis* HMTZ24 using MGEfinder v1.0.

MGE	Type	Contig / Position	Sequence Identity	No. Substitutions	E-value	Accession No.
Tn6009	Integrative Conjugative Element	49 / 13895-15783	99.95%	1	0.0	EU399632
ISLgar5	Insertion sequence	4 / 61430-62765	99.18%	11	0.0	AKFO01000017.1

A total of 14 virulence factor genes were identified (Table 5). Six of these are associated with biofilm formation: three pili genes (*ebpA, ebpB, ebpC*), collagen adhesion (*ace*), sortase-assembled pili (*strA*), and enterococcal surface protein (espfs). Other virulence genes include those linked to sex hormone response (*cad, camE, cCf10, cOB1*), gelatinase (*gelE*), oxidative stress resistance (*tpx*), endocarditis antigen (*efaA*), and macrophage persistence (*ElrA*).

**Table 5:** Virulence factor genes identified in the genome of *E. faecalis* HMTZ24 using VirulenceFinder v2.0.

Virulence factor	Identity (%)	Query / template length	Contig Number	Position in contig	Accession number
*ebpA*	99.34	3312 / 3312	Contig_20	4290-7601	CP002621.1
*ebpB*	99.3	1431 / 1431	Contig_20	2856-4286	CP003726.1
*ebpC*	99.63	1884 / 1884	Contig_20	976..2859	CP002491.1
*ace*	98.88	1158 / 1743	Contig_37	585-1742	AF260878.1
*SrtA*	100	735 / 735	Contig_47	4598-5332	AE016830.1
*espfs*	99.79	2385 / 5622	Contig_75	3681-6065	AF034779.1
*cad*	100	930 / 930	Contig_74	2411-3340	CP002621.1
*camE*	99.8	501 / 501	Contig_6	69591-70091	AF435442.1
*cCF10*	100	828 / 828	Contig_8	65572-66399	CP002491.1
*cOB1*	99.88	819 / 819	Contig_51	7707-8525	CP002621.1
*gelE*	99.93	1530 / 1530	Contig_13	14456-15985	CP002491.1
*tpx*	99.8	510 / 510	Contig_41	3164-3673	FP929058.1
*efaAfs*	99.68	927 / 927	Contig_14	27867-28793	FP929058.1
*ElrA*	99.59	2172 / 2172	Contig_30	3199-5370	CP002621.1

## 4 Experimental Design, Materials and Methods

*Enterococcus faecalis* HMTZ24 was cultured on sheep blood agar, and single colonies were transferred to mannitol salt agar (HiMedia, India). Colonies that fermented mannitol, Gram-positive, and catalase-negative [4] were subsequently selected for further analysis. The catalase test was performed by transferring a small amount of bacterial growth onto a glass slide, followed by the addition of one drop of 3 % hydrogen peroxide (H_2_O_2_). The absence of visible bubble formation after adding H_2_O_2_ indicated that the isolate was catalase-negative. Genomic DNA (gDNA) was isolated from pure colonies of *E. faecalis* HMTZ24, grown on mannitol salt agar (HiMedia, India) for 24 h at 37°C using the Bacterial DNA Isolation Kit (Foregene Co., Ltd, China) according to manufacturer’s instructions. The concentration and the purity of the gDNA were measured using a NanoDrop 2000 spectrophotometer (Thermo Scientific, USA).

The genomic DNA library was prepared using the TruSeq Nano DNA Library kit (350 bp insert size), and whole-genome sequencing was performed on the Illumina NovaSeq 6000 platform with 2 × 150 bp paired-end reads at Macrogen Inc. (Seoul, South Korea). Raw reads were quality-filtered and adapter sequences were removed using Trimmomatic v0.30 with default settings [5]. The filtered reads were assembled with SPAdes v3.5 [6] using k-mer lengths of 21, 33 and 55. Genome annotation was performed through the NCBI Prokaryotic Genome Annotation Pipeline (PGAP) v6.9 [7]. Genome assembly statistics were obtained with QUAST v5.3.0 software [8], and completeness was assessed with CheckM v1.2.3 [9].

Phylogenomic analysis and digital DNA–DNA hybridization (dDDH) were carried out using the Type Strain Genome Server (TYGS) [10]. Average nucleotide identity (ANI) between *E. faecalis* HMTZ24 and *E. faecalis* NBRC 100480 was calculated using the OrthoANIu tool [11]. Multilocus sequence typing (MLST) and allele profiling were performed using the PubMLST database [12]. Antimicrobial resistance genes were predicted using both the Comprehensive Antibiotic Resistance Database (CARD) v4.0.1 [13] and ResFinder v4.7.2 [14] each run with default settings. For ResFinder, a 90 % identity threshold, a minimum hit length of 80 %, and *Enterococcus faecalis* as the reference species were applied. Mobile genetic elements (MGEs) were identified with MGEfinder v1.0.3 [15]. Virulence factor genes were predicted using VirulenceFinder v2.0 [16], with thresholds set at 90 % sequence identity and 80 % minimum gene length.

## Limitations

A limitation of this study is that the genome sequence is incomplete and fragmented (128 contigs), which may impact the accuracy and completeness of the analyzed data*.*

## Ethics Statement

Ethical approval was obtained from the Institutional Ethics Committee of the College of Science, University of Mosul. The protocol code for this study is 45E-228, dated September 16, 2024. Written informed consent was obtained from the patient in accordance with the Declaration of Helsinki 1964*.*

## Credit Author Statement

**Talal S. Salih:** Conceptualization, Methodology, Formal Analysis, Writing, Original draft preparation; **Zeyad T. Al-Rrassam:** Conceptualization, Data curation, Writing- Reviewing and Editing; **Muhammad A. Muhammad:** Investigation, Formal analysis, Writing - Original Draft; **Hozan R. Ziwar:** Conceptualization, Data Curation, Validation, Resources, Writing; **Mohammed M. Mohammed:** Conceptualization, Methodology, Validation, Resources, Writing.

## Data Availability

Enterococcus faecalis strain HMTZ24, whole genome shotgun sequencing project (Original data).
